# Human Coronaviruses Associated with Upper Respiratory Tract Infections in Three Rural Areas of Ghana

**DOI:** 10.1371/journal.pone.0099782

**Published:** 2014-07-31

**Authors:** Michael Owusu, Augustina Annan, Victor Max Corman, Richard Larbi, Priscilla Anti, Jan Felix Drexler, Olivia Agbenyega, Yaw Adu-Sarkodie, Christian Drosten

**Affiliations:** 1 Kumasi Centre for Collaborative Research in Tropical Medicine, Kwame Nkrumah University of Science and Technology, Kumasi, Ghana; 2 Institute of Virology, University of Bonn Medical Centre, Bonn, Germany; 3 Institute of Renewable and Natural Resources, Kwame Nkrumah University of Science and Technology, Kumasi, Ghana; 4 Department of Clinical Microbiology, Kwame Nkrumah University of Science and Technology, Kumasi, Ghana; Faculty of Biochemistry Biophysics and Biotechnology, Jagiellonian University, Poland

## Abstract

**Background:**

Acute respiratory tract infections (ARI) are the leading cause of morbidity and mortality in developing countries, especially in Africa. This study sought to determine whether human coronaviruses (HCoVs) are associated with upper respiratory tract infections among older children and adults in Ghana.

**Methods:**

We conducted a case control study among older children and adults in three rural areas of Ghana using asymptomatic subjects as controls. Nasal/Nasopharyngeal swabs were tested for Middle East respiratory syndrome coronavirus (MERS-CoV), HCoV-22E, HCoV-OC43, HCoV-NL63 and HCoV-HKU1 using Reverse Transcriptase Real-Time Polymerase Chain Reaction.

**Results:**

Out of 1,213 subjects recruited, 150 (12.4%) were positive for one or more viruses. Of these, single virus detections occurred in 146 subjects (12.0%) and multiple detections occurred in 4 (0.3%). Compared with control subjects, infections with HCoV-229E (OR = 5.15, 95%CI = 2.24–11.78), HCoV-OC43 (OR = 6.16, 95%CI = 1.77–21.65) and combine HCoVs (OR = 2.36, 95%CI = 1.5 = 3.72) were associated with upper respiratory tract infections. HCoVs were found to be seasonally dependent with significant detections in the harmattan season (mainly HCoV-229E) and wet season (mainly HCoV-NL63). A comparison of the obtained sequences resulted in no differences to sequences already published in GenBank.

**Conclusion:**

HCoVs could play significant role in causing upper respiratory tract infections among adults and older children in rural areas of Ghana.

## Background

Acute respiratory tract infections (ARI) are a leading cause of morbidity and mortality among young children and adults in developing countries, especially Africa [1]–[3]. The majority of ARI are of viral origin including respiratory syncytial virus (RSV), Influenza viruses, Rhinoviruses, Parainfluenza viruses, Human metapneumovirus and human coronaviruses (HCoVs) [4]–[7]. Coronaviruses (CoVs) are enveloped RNA viruses and belong to the family *Coronaviridae* and subfamily *Coronavirinae*. CoVs are positive strand viruses with genome sizes ranging from 27–33 kilobases (kb). They are classified into four genera named *Alphacoronavirus*, *Betacoronavirus*, *Gammacoronavirus* and *Deltacoronavirus* [8]. HCoV-NL63 and HCoV-229E belong to the alphacoronaviruses while HCoV-OC43, HCoV-HKU1, and the severe acute respiratory syndrome coronavirus (SARS-CoV) belong to the betacoronaviruses [8]. A recent HCoV that was first identified in the Middle East region is termed Middle East respiratory syndrome coronavirus (MERS-CoV) and belongs to the genus *Betacoronavirus* [9]. Despite the detection of MERS-CoV-related sequences in bats worldwide and neutralising antibodies against MERS-CoV in camels, the evolutionary source of this novel HCoV still remains unclear [10]–[17].

The evolving trend in the emergence of new strains of HCoVs coupled with an increase in advanced molecular techniques has revived the interest of researchers in finding the epidemiologic association between respiratory diseases and HCoV infection. Some studies in developed countries have reported HCoV-OC43 and HCoV-229E to be responsible for up to 10% of upper respiratory tract infections [18], [19] whereas others identified these viruses along with HCoV-NL63 and HCoV-HKU1 to be more prevalent in severe respiratory tract infections of immunocompromised individuals, institutional elderly subjects and infants [20]–[23]. Severe infections and deaths have been associated with MERS-CoV infections [17], [24]–[27]. Other studies on the contrary identified HCoVs to be quite common in healthy individuals thus raising questions about its association with respiratory illness [28], [29]. Finally, even though HCoVs are believed to be most relevant in young children, the contribution of these viruses to disease in older children and adults still needs to be addressed worldwide [30].

Knowledge of the contribution of HCoVs to the burden of respiratory tract infections in rural areas of Africa is rare. Rural regions are considered as the focus of morbidity and mortality associated with infectious diseases due to the poor hygienic practices and the lack of quality healthcare systems. Information on the aetiologies of disease in these areas will therefore help to reduce disease burden. However, even in developed countries most studies are hospital based and lack healthy humans as control groups [6], [7], [31].

This study aims to provide information on the association of HCoVs with upper respiratory tract infections in remote rural areas of Ghana with additional focus on seasonal variation and the spectrum of circulating HCoV strains.

## Materials and Methods

### Ethics statement

The study protocol was approved by the Committee for Human Research, Publications and Ethics of KATH and School of Medical Sciences, KNUST, Kumasi, Ashanti region, Ghana (Ethical clearance reference: CHRPE 49/09). We first explained the study protocol captured on the “Participant Information Leaflet and Consent Form” to every subject who was enrolled in the study. This was done in the local language of the subjects. Once they agreed, we asked them to thumbprint the consent form attached to the participant leaflet form for subjects who were illiterates. For those who were literates, we asked them to sign the consent forms.

Written informed consent was obtained for data and sample collection for all subjects. For subjects less than 18 years of age, written informed consent was obtained from parents or guardians and assent from the minors.

### Study Area

**Figure 1:**
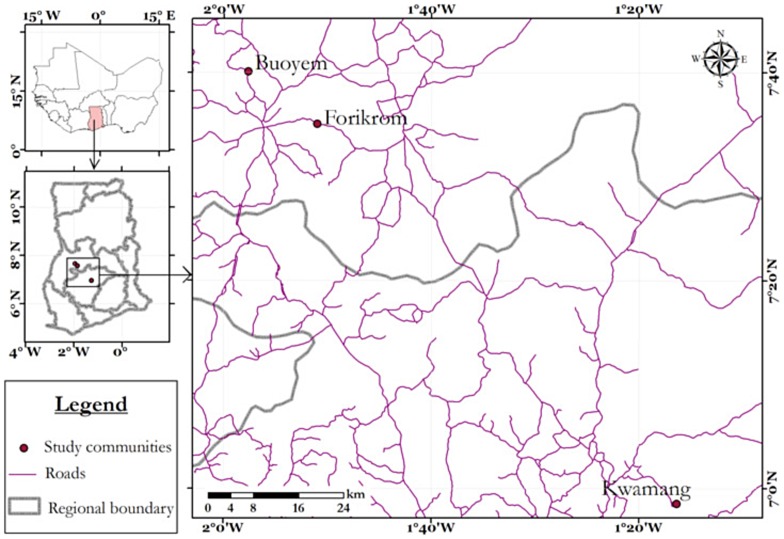
Geographical location of study areas in Ghana. The study communities are represented by three red dots. The geographic coordinates on the horizontal and vertical regions of the bar show the latitude and longitude coordinates. The red lines show the roads the link the respectively communities.

The study was performed in three rural areas of Ghana, namely Buoyem, Kwamang and Forikrom communities, from September 2011 to September 2012. **Figure 1** shows the geographic location of all three communities. Buoyem and Forikrom communities are located in the Techiman municipality of the Brong Ahafo Region of Ghana. The Techiman municipality is among the 22 administrative districts in the region [32]. It shares common boundaries with the Wenchi district to the northwest and Nkoranza district to the southeast. The municipality has a total land surface of 669.7 Km^2^ with climate and vegetation that promotes the production of food. Kwamang community is in the Sekyere Central district of the Ashanti Region of Ghana. The community is part of 5 sub-districts of Sekyere Central and forms one third of the total land size of the district [32].

### Population Characteristics

The population of Techiman municipality based on the 2010 population census is 206,856 [33]. The projected population of Forikrom and Buoyem are 3,800 and 3,900 individuals respectively [34]. The Techiman municipality has a population density of 318 people per Km^2^ with several ethnic groups [32]. The population of Sekyere Central district is 71,232 and the projected population of Kwamang is 8,000 [34]. The population density of the district is 64 people per Km^2^ [35].

### Study design

We conducted an unmatched case control study to identify the association between HCoVs and upper respiratory tract infection. Subjects were identified as cases if they presented with symptoms of upper respiratory illness with rapid onset of any of the following conditions: cough, sneezing, runny nose and nasal congestion. Subjects were selected as controls if they did not present with any symptom of upper respiratory illness for at least 8 days prior to recruitment. Subjects were excluded if they were less than 10 years old, had a history of epistaxis or refused clinical samples to be collected.

For a random selection of the study participants, the communities were divided into four quadrants using major roads. Social centres at each quadrant were selected at random and every other adjacent house starting from the social centre was marked and subjects were selected. Once the communities got well informed about the study, radio announcements were used to call individuals to major community centres where recruitment was continued.

Recruitment of subjects was done on seasonal basis. There are two major seasons in the study areas; the wet and harmattan season. The harmattan is characterised by dry wind carrying dust from the Sahara desert while the wet has mainly heavy rains with high humidity. The periods in-between these seasons (pre-harmattan and interim) are characterised by hot temperatures and minor rains. Major recruitments were made in the pre-harmattan season (September, October, November), harmattan season (December, January, February) and wet season (May, June, July). Minor sampling was done in the pre- wet season (March, April). Cases were recruited in each season alongside controls. Demographic information was collected from all subjects using structured questionnaires. The questionnaires were written in English and interpreted to subjects in their local dialects.

### Clinical Sampling

Nasopharyngeal specimens were taken with flocked swabs (Copan, Italy) from subjects by gently inserting the swab up the nostril towards the pharynx until resistance was felt and was then rotated 3 times to obtain epithelial cells. In cases where subjects objected to nasopharyngeal sampling, nasal swabs were taken. From field observations, nasopharyngeal swabs were taken from about 90% of all study subjects. The swabs were stored in 1.5 ml RNAlater (Qiagen, Hilden, Germany) and transported to the laboratory where they were frozen at −20°C until testing.

### Laboratory Methods

Viral RNA was extracted from the samples using the spin protocol of the QIAamp Viral RNA Mini Kit (Qiagen, Hilden, Germany) as described by the manufacturer and extended with an additional centrifugation step for 5 min at 20,000×g to dispose all residues of washing buffer before elution. Samples were eluted in 100 µl of pre-warmed 80°C AVE buffer.

Real-time reverse transcription polymerase chain reaction (RT-PCR) was performed for the detection of five HCoVs; HCoV-229E, HCoV-OC43, HCoV-NL63, HCoV-HKU1 and MERS-CoV. Testing of samples was performed by pooling in batches of five to ten samples. Singleplex RT-PCR was used for detection of MERS-CoV as described elsewhere [36] while the other four HCoVs were detected with multiplex RT-PCR.

For Multiplex RT-PCR testing of HCoV-NL63 and HCoV-HKU1, a 25 µl reaction was set up containing 5 µl of RNA, 1 µl of 10 mM dNTP mix (Qiagen), 5 µl of OneStep 5x buffer (Qiagen), 1 µl of Enzyme Mix (Qiagen), 600 nM of each forward and reverse primers for HCoV-NL63, 400 nM of each forward and reverse primers for HCoV-HKU1, 200 nM probe for each virus and 7 µl of RNASE free water. HCoV-229E and HCoV-OC43 multiplex RT-PCR testing was similarly performed as described above except that 400 nM of each forward and reverse primers for HCoV-229E and HCoV-OC43 and 200 nM of the respective probes were used. The amplification procedure comprised reverse transcription at 55°C for 20 min followed by initial denaturation at 94°C for 3 min and 45 cycles of 94°C for 15 s and 58°C for 30 s.

The sequences of primers and probes for each assay are shown in **Table 1** as described elsewhere [18], [29], [36]. All PCR’s were performed using light cycler 480 II (Roche, Germany).

**Table 1:** Primers used for PCR Testing.

Virus Type	Forward Primers	Reverse Primer	Probe	Target Region	Usage	Reference
	5′–––––>3′	5′––––––>3′				
HCoV-229E	CAGTCAAATGGGCTGATGCA	AAAGGGCTATAAA GAGAATAAGGTATTCT	JOE-CCCTGACGACCACGTTGTGGTTCA-BHQ1	Nucleoprotein	RT-PCR	[18]
	F1-GTGCTTAGTCTTGTTAGGAGTGG	TCACGAACTGTCTTAGGTAGTGC	N/A	Spike	Sequencing	This study
	F2-GTAAGTTGCTTGTAAGGGGTAATG		N/A	Spike	Sequencing	This study
HCoV-OC43	CGATGAGGCTAT TCCGACTAGGT	CCTTCCTGAGCCTT CAATATAGTAACC	FAM-TCCGCCTGGCACGGTACTCCCT-BHQ1	Necleoprotein	RT-PCR	[18]
	F1-ACTAGGCTGCATGATGCTTAGA	CACATATTATACTGGCAAACAGA	N/A	Spike	Sequencing	This study
	F2-GCATGATGCTTAGACCATAATCT		N/A	Spike	Sequencing	This study
HCoV-NL63	GACCAAAGCACTGAATAACATTTTCC	ACCTAATAAGCCTC TTTCTCAACCC	FAM-ATGTTATTCAGTGCTTTGGTCCTCGTGAT-BHQ1	Necleoprotein	RT-PCR	[29]
	F1-GTGTGGTGACATTCACAGTAACG	GTGTGGTGACATTCACAGTAACG	N/A	Spike	Sequencing	This study
	F2-GAGTTTGATTAAGAGTGGTAGGT		N/A	Spike	Sequencing	This study
HCoV-HKU1	CCTTGCGAATGAATGTGCT	TTGCATCACCACTG CTAGTACCAC	JOE-TGTGTGGCGGTTGCTATTATGTTAAGCCTG-BHQ1	Replicase 1b	RT-PCR	[29]
	F1-TTGCCTACAACATTAGCTGTTA	CCACGTTCTTGATA AAAATGAAAATAC	N/A	Spike	Sequencing	This study
	F2-CAACATTAGCTGTTATAGGTGAT		N/A	Spike	Sequencing	This study
MERS-CoV	GCAACGCGCGATTCAGTT	GCCTCTACACGGGACCCATA	FAM-CTCTTCACATAATCGC CCCGAGCTCG-TAMRA	Envelope	RT-PCR	[36]

F1 and F2: First and second round forward primers for sequencing of samples, one reverse primer for both rounds.

NA: Not applicable.

### Controls and Standards

All runs included RNase free water as a negative control and quantified in-vitro transcripts of each virus as positive control. The in-vitro transcripts were prepared as described previously [36]. Briefly, target amplicons were TA cloned. Plasmids were purified and reamplified using vector specific oligonucleotides and then finally *in vitro* transcribed using a T7 promoter (Ambion Megascript kit, Invitrogen). Ten-fold dilutions of the RNA transcripts were prepared using RNase free water containing 10 µg/ml of carrier RNA. Standard curves were generated from serial dilutions of the in-vitro transcripts. Two controls with concentrations of 10^2^ and 10^3^ copies per reaction were included in each PCR run for HCoV-NL63, HCoV-OC43 and HCoV-HKU1. For MERS-CoV assay, concentrations of 10^1^ and 10^2^ copies per reaction were used. All positive samples were quantified using the positive controls and connected external standard curves.

### Sequencing of positive HCoVs using Heminested PCR

For all positive samples, PCRs were done to amplify the first 500 base pairs of the spike gene using primers listed in **Table 1**. For the first round of PCR, a 25 µl reaction was set up containing 5 µl of RNA, 12.5 µl of 2 X reaction buffer (Invitrogen; containing 0.4 mM of each dNTP and 3.2 mM MgSO4), 1 µl of reverse transcriptase/Taq mixture from the kit, 0.4 µl of a 50 mM MgSO_4_, 1 µg of non-acetylated bovine serum albumin (Roche) and 400 nM of each primer. The amplification procedure comprised 15 min at 50°C; 3 min at 95°C; 10 cycles of 15 s at 94°C, 15 s starting at 60°C with a decrease of 0.5°C per cycle, and 40 s at 72°C; and 40 cycles of 15 s at 95°C, 30 s at 56°C, and 40 s at 72°C. A second-round reaction was set up from the first round using 5 µl of 10 X reaction buffer (Invitrogen), 2.5 µl of a 50 mM MgCl_2_ solution, 200 nM of dNTP, 400 nM of each forward and reverse primer and 0.2 µl of platinum Taq DNA Polymerase. Thermal cycling was performed at 95°C for 3 min and 45 cycles of 95°C for 15 s, 56°C for 15 s and 72°C for 40 s, followed by a 2 min extension step at 72°C. All obtained PCR products were sequenced and compared to GenBank via the BLAST Algorithm as well as aligned together with reference sequences from the GenBank.

### Data Analysis

All data obtained from the communities were recorded using EPI INFO version 5 (CDC, Atlanta) and imported into Microsoft Excel. Subsequent analysis was performed using R statistical software version 2.15.2 [37]. Categorical variables and their association with respiratory agents were analysed using the Fischer’s exact test. Continuous variables were expressed as medians with their inter-quartile ranges (IQR). A non-parametric K-sample test on the equality of medians was used to evaluate the differences in the medians of the various subgroups of the continuous variables. The association of HCoVs with upper respiratory tract infection was assessed by fitting five logistic regression models controlling for age group, age as a continuous variable and study communities. Results were expressed as adjusted odd ratios and 95% confidence interval (CI). Graphs were plotted using ggplot2 package [38] and R base plots. For all analysis, a two-sided p-value of less than 0.05 was considered significant.

## Results

### Reproducibility of HCoV assays

To determine the reproducibility of the human Coronavirus RT-PCR assays used in this study, we included two in-vitro transcripts with a defined RNA concentration of 100 copies per reaction in each RT-PCR run. The mean and standard deviations specified as m (s.d) of the real-time PCR signal crossing points for HCoV-HKU1, HCoV-NL63, HCoV-OC43, MERS-CoV and HCoV-229E were respectively determined to be 31.27 (0.24), 33.61 (0.34), 35.06 (0.6), 33.85 (0.94) and 36.31 (1.12). It was concluded that all assays provided high sensitivity and reliable performance.

### Characteristics of Study Subjects

A total of 1213 subjects were recruited during the study period. Major recruitments were done in the pre-harmattan (385, 32%), harmattan (215, 18%) and wet seasons (516, 43%), and minor recruitment was done in the pre-wet season (97, 8%). Overall four 487 (40%) were enrolled in 2011 and 726 (60%) were enrolled in 2012. Of the 1213 subjects recruited, 620 (51.1%) were controls and 593 (48.9%) were cases. Table 2 shows the demographic description of cases and controls. The occupation of subjects was classified into one group with a high rate of social interactions and a second group with a lesser rate of interactions. Occupations with high social contact include health workers, dressmakers, students, teachers, hairdressers, traders, drivers and food vendors. The second group comprised farmers, hunters, traditional authorities and carpenters.

**Table 2:** Demographic characteristics of study subjects.

	Control groups	Case groups
Variables	n = 620	n = 593
**Study communities**		
Buoyem n (%)	194 (31.3)	208 (35.1)
Forikrom n (%)	162 (26.1)	195 (32.9)
Kwamang n (%)	264 (42.6)	190 (32)
**Gender (Female) n (%)**	360 (58.2)	334 (56.3)
**Age n % (IQR)**	40 (24.5–54)	35 (20–52)
**Occupation**		
High social contact n (%)	260 (41.9)	264 (44.5)
Low social contact n (%)	360 (58.1)	329 (55.5)
**Type of Accommodation**		
Crowded accommodation n (%)	408 (66.2)	397 (67.7)
Non-crowded accommodation n (%)	208 (33.8)	189 (32.3)

### Human Coronavirus Epidemiology

The present study identified four HCoVs (HCoV-229E, HCoV-OC43, HCoV-NL63 and HCoV-HKU1). No sample was positive for the recently emerged MERS-CoV. The overall detection of HCoVs was 12.4% (150/1213). Single virus detections occurred in 146 samples (12.0%) and multiple detections occurred in 4 samples (0.3%). The most prevalent virus identified was HCoV-NL63 (82 detections, 6.8%) followed by HCoV-229E (41, 3.4%), HCoV-OC43 (18, 1.5%) then HCoV-HKU1 (5, 0.4%). Three samples were positive (0.2%) for HCoV-OC43 and HCoV-229E and one sample for HCoV-NL63 and HCoV-229E.

### Virus distribution in cases and controls

**Figure 2:**
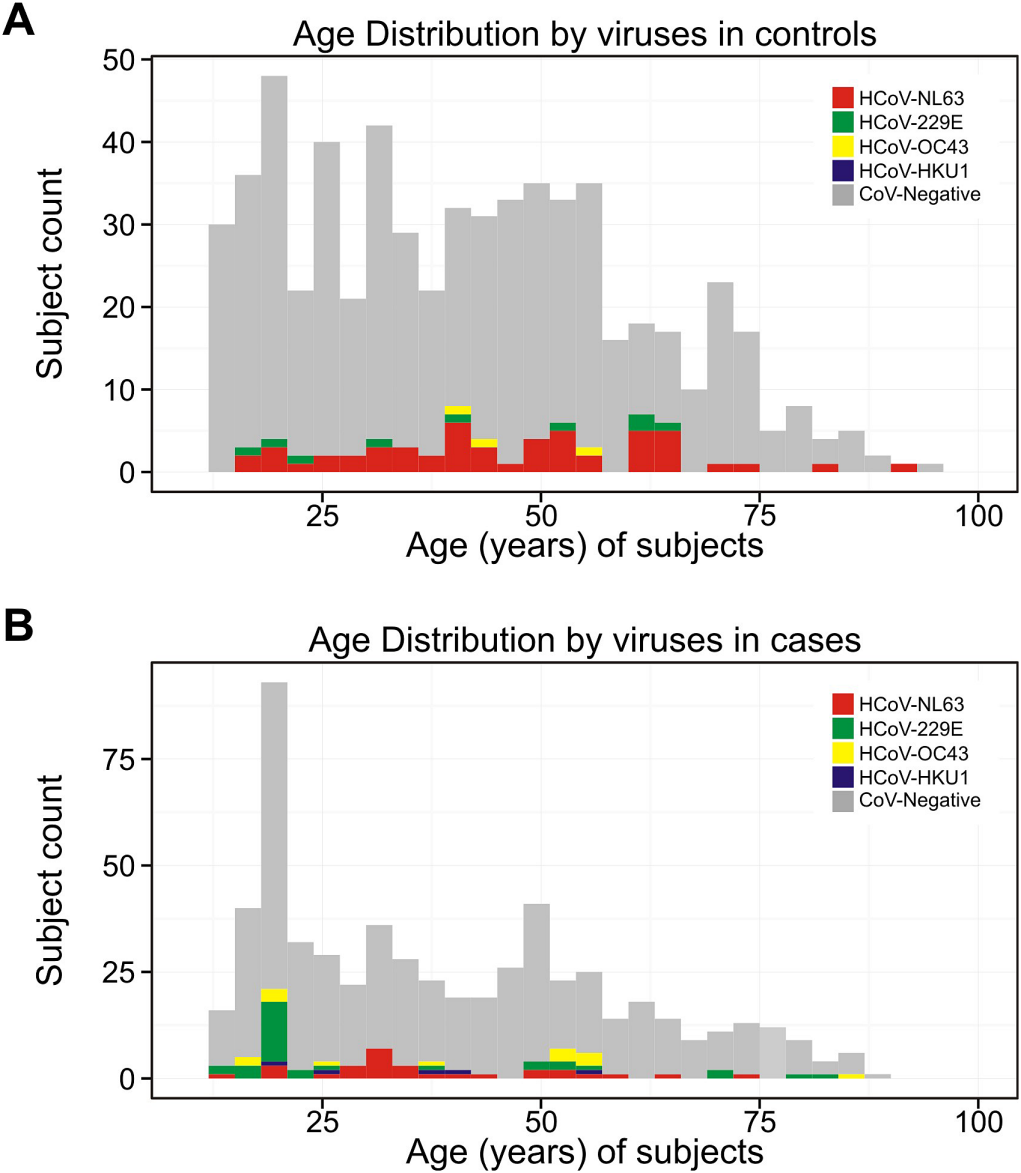
Virus distribution among age categories of cases and controls. The x-axis represents the age groups and y-axis shows the number of subjects. Grey bars represent the total number of samples per age group, coloured bars the virus-positive samples. Colour coding is according to virus species as indicated in the legend.

As shown in **Figure 2**, a comparison of the age distribution of the different viruses detected in case and control groups revealed HCoV-229E to be more frequent in cases with age below 20 years whereas HCoV-NL63 was common in the middle age group. Among control subjects, HCoV-NL63 was found to occur almost equally in all ages. We did not find a significant difference (p = 0.43) comparing overall virus detection rates in all subjects with ages from 10 to 40 years (54.1%; 84/146) and those above 40 years (61%; 42.1).

HCoVs were detected in 65 (10.5%; 65/629) controls and 81 (13.7%; 81/593) cases. Notably, this difference was not statistically significant (p = 0.11). The most common virus among cases and controls were HCoV-229E (36, 6.1%) and HCoV-NL63 (53, 8.5%), respectively (**Table 3**). HCoV-229E and HCoV-OC43 were more frequently encountered in cases than in controls whereas HCoV-NL63 was more common in control subjects. The results for all virus detections in cases and controls did not change significantly when cases showing co-infections with multiple CoV were excluded from the analysis.

**Table 3:** Viruses detected in cases and controls.

Viruses	Control groups	Case groups	Total	p-value
	n = 620	n = 593	n = 1213	
HCoV-229E [n (%)]	9 (1.5)	36 (6.1)	45 (3.7)	**0.001**
HCoV-HKU1 [n (%)]	0 (0)	5 (0.8)	5 (0.4)	0.065
HCoV-NL63 [n (%)]	53 (8.5)	30 (5.1)	83 (6.8)	**0.022**
HCoV-OC43 [n (%)]	3 (0.5)	18 (3)	21 (1.7)	**0.001**
Overall HCoV [n (%)]	65 (10.5)	81 (13.7)	146 (12)	0.107

High HCoV detection rates in control subjects were compatible with prolonged excretion of very low levels of viral RNA after convalescence. As these detections would reduce the clinical utility of RT-PCR diagnostics, the analysis was repeated excluding all cases showing viral loads of 100 or less copies per reaction. The modified dataset is presented in **Table 4**. Except for HCoV-NL63 and -HKU1, virus detection rates were now higher in cases as compared to controls. Also the overall detection rate for all HCoVs including HCoV-NL63 and -HKU1 was higher among cases compared to control subjects (p = 0.001).

**Table 4:** Viruses detected in concentrations above 100 copies per RT-PCR reaction.

Viruses	Control group	Case group	Total	p-value
	n = 620	n = 593	1213	
HCoV-229E n (%)	7 (1.1)	35 (5.9)	42 (3.5)	**0.001**
HCoV-HKU1 n (%)	0 (0)	3 (0.5)	3 (0.2)	0.23
HCoV-NL63 n (%)	20 (3.2)	20 (3.4)	40 (3.3)	1
HCoV-OC43 n (%)	3 (0.5)	17(2.9)	20 (1.6)	**0.002**
Overall HCoVs n (%)	30 (4.8)	68 (11.5)	98 (8.1)	**0.001**

**Figure 3:**
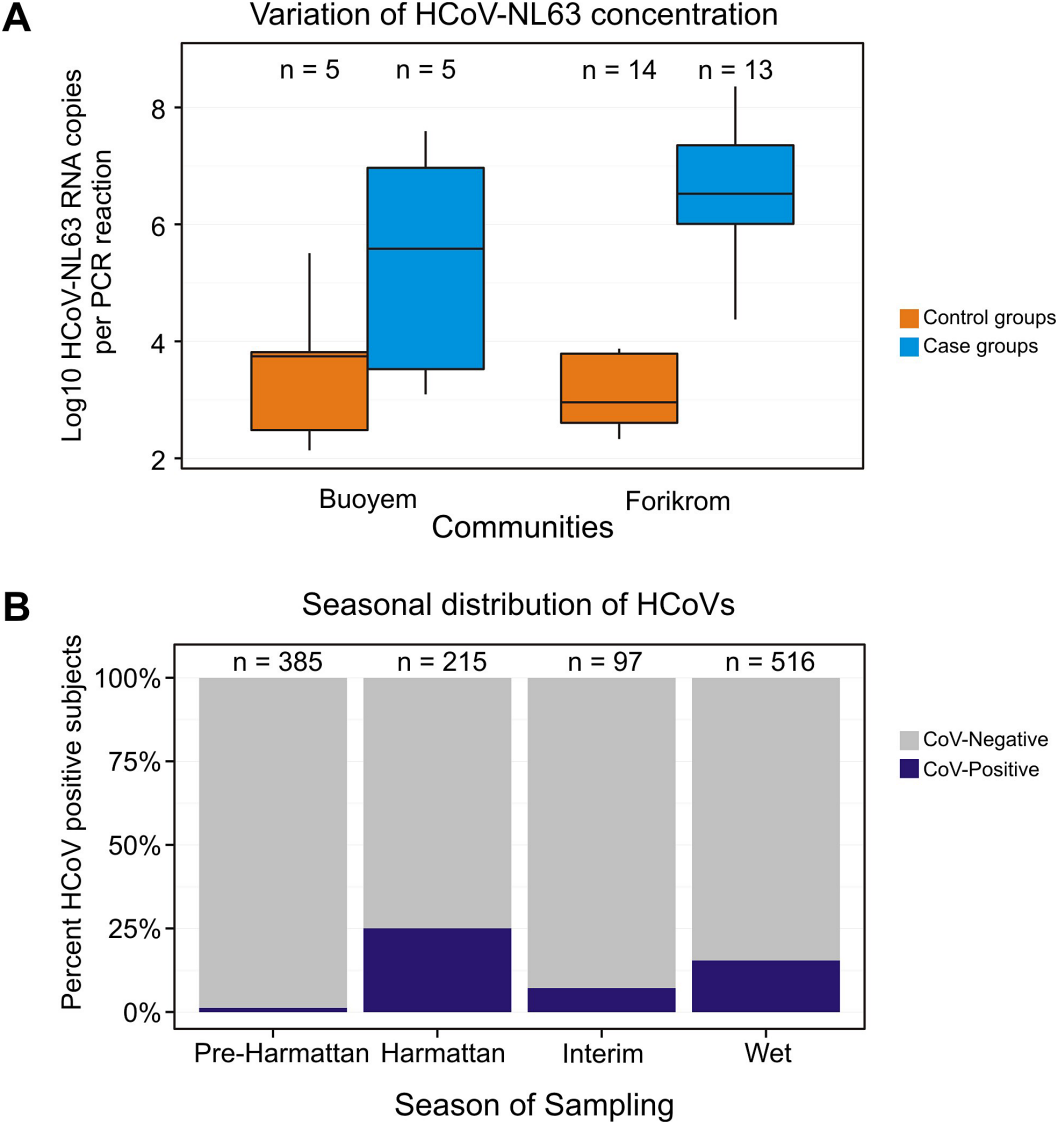
Seasonal distribution and concentration variation of HCoVs. A: Variation of HCoV-NL63 Concentration. The study areas are represented on the x-axis and the y-axis shows the log10 of HCoV-NL63 concentration in copies per RT-PCR reaction. Data for Kwamang is not shown because of the low numbers detected. Significant differences in viral loads between healthy and control subjects were detected for only the Forikrom community. B: Seasonal distribution of HCoVs. The x-axis shows the various seasons and the “n” value on top of each bar represents the number of subjects recruited in each season. The y-axis shows the percentage of subjects positive for HCoVs.

Using the dataset from which cases containing less than 100 copies per reaction had been eliminated, quantitative results were compared between cases and controls. Virus loads of HCoV-229E showed no significant difference between cases and controls. Cases yielded an average of 27,400 RNA copies per RT-PCR reaction (interquartile range (IQR) 6.4×10^2^−2.4×10^7^) while controls had 197,000 copies per PCR reaction on average (IQR = 4110–1.28×10^6^). The trend was similar for cases and controls with HCoV-OC43 detection (115,000 copies per PCR reaction; IQR = 412–7.5×10^5^ vs. 5,480 copies per PCR reactions; IQR = 3270–3.47×10^7^). HCoV-NL63 median concentration was however significantly higher in cases compared to controls (2.41×10^6^ copies per PCR reaction; IQR = 1.96×10^4^−2.3×10^6^ vs. 1,876.5 copies per PCR reaction; IQR  = 387.2–8.6×10^4^, p = 0.003). Low detection rates for HCoV-HKU1 did not warrant a quantitative comparison for this virus. Due to the differences in HCoV-NL63 virus loads, the virus load of cases and controls were analysed in different communities. **Figure 3** **A** shows that there was a significant difference (higher in cases compared to controls) in Forikrom (p = 0.005) but not in the other study areas.

### Seasonal distribution of HCoVs

The present study also investigated the seasonal variation of HCoVs in all study communities (**Figure 3** **B**). High proportions of HCoVs were identified in the harmattan season (54/215, 25.1%) compared to the wet (80/516, 15.5%) seasons (p = 0.003). The increase of detection of HCoVs in the harmattan season was also significant compared to detections in the pre-harmattan season (1.3%, p<0.01) and the interim season (7.2%, p<0.0004). The most frequent viruses detected in the harmattan and wet seasons were HCoV-229E and HCoV-NL63, respectively. HCoV-OC43 and HCoV-HKU1 were almost equally distributed throughout the year.

To further explore the frequency of virus circulation among subjects with high or low social interactions, all subjects consisting of cases and controls were regrouped into low social contact groups (689/1,213; 56.8%) and high social contact groups 524;(43.2%) based on their occupations. HCoVs were identified in 88 (12.8%) individuals in the low social contact group and 58 (11.1%) in the high social contact group. There was no significant difference (p = 0.42) in the proportion of the overall HCoVs detected in the two groups. A comparison of the individual HCoVs however showed high proportions of HCoV-229E in the high social contact group (26/524; 5.0%) compared to the low social contact group (15/689; 2.2%). The difference in proportions of HCoV-229E was significant (p = 0.01). All other HCoV detections were not significant between the groups except HCoV-NL63, which was slightly higher (p = 0.04) in the low social group (56/689; 8.1%) compared to the high social group (26/524; 5.0%).

### Sequence typing

To determine whether HCoVs circulating in rural Africa were similar to viruses encountered in the northern hemisphere, a representative sample of the encountered viruses were sequenced. Sequencing of a variable part of the viral spike protein gene was successful for 53 out of 146 samples (36.3%). Of the 53, 12 (22.6%) were HCoV-OC43, 14 (26.4%) were HCoV-NL63, 24 (45.3%) were HCoV-229e and 3 (5.7%) were HCoV-HKU1. A comparison of the all sequences to sequences published in GenBank identified no relevant differences against previously described viruses. In contrast, sequences resembling the homologous spike gene portions of bat-associated HCoV-229E-related coronaviruses previously described by our group in animals sampled in the study area were not detected in any sample [39], [40]. All sequences have been deposited in the GenBank under the accession numbers KJ768612–KJ768646 and KJ796450–KJ796467.

## Discussion

HCoVs contribute significantly to the burden of upper respiratory tract infections. Therefore most developed countries have investigated the molecular and epidemiological profile of these viruses. On the contrary, information from developing countries, particularly in tropical areas of sub-Saharan Africa is scarce. The few published data were mostly from hospitalised subjects under five years and without the inclusion of control subjects. We performed a case-control study in sub-Saharan Africa that reports the detection of HCoVs in older children and adults with and without upper respiratory tract infection.

Our study resulted in the detection of 146 (12.4% of tested specimens) HCoVs occurring in both cases and controls. A comparison of viruses in cases and controls showed higher detections of HCoV-229E and HCoV-OC43 among cases compared to controls. These findings are similar to previous reports that identified HCoV-229E and HCoV-OC43 as being commonly associated with upper respiratory tract infections [18], [41]. These differences did not change significantly when low viral loads of less than 100 copies per RT-PCR reaction were excluded from the analysis. Robustness of results against the exclusion of low-copy samples is important, as RT-PCR suffers from inconsistencies at the lower end of the detection range, in particular if carried out in settings with only a basic level of training and technology, such as in this study. Caveats include the potential of contamination of test tubes with low copy numbers of target RNA template. Moreover, test sensitivity can vary greatly at the lower end of the detection range, which is why we have demonstrated for our methodology that more useful results are achieved at levels above 100 copies per reaction.

In this light it is interesting that HCoV-NL63 was found to occur at almost equal rates in cases and controls. This observation was previously reported among young children [28] and could possibly suggest the virus may have an insignificant role in causing ARI among adults and older children. However, our results have to be interpreted with caution because some control subjects positive for HCoV-NL63 might have had yet subclinical infections at the time of recruitment. This scenario cannot be ruled out, especially because HCoV-NL63 has generally been associated with mild respiratory infection. Lower levels of virus replication in controls as opposed to cases are also suggested by the diverging levels of virus concentration particularly for HCoV-NL63.

One limitation of this study could be the overestimation of viral detection rates in control subjects probably due to shedding of HCoVs following earlier symptomatic infections. The ideal approach would have been to recruit controls who had no symptoms of respiratory infection for at least one month. However the enrolment of such subjects during sampling will be difficult, especially in rural areas, where illiteracy rate is high and subjects may not be able to document or recall the occurrence of symptoms for prolonged periods. A further explanation of this overestimation might be the persistent or residual RNA fragments in the respiratory tract of adults. This phenomenon of RNA persistence was generally described for other respiratory viruses in children [42]–[44]. Another limitation was our inability to test other known respiratory viruses among our study subjects. Future studies could explore the role of other viruses among subject with URTI compare to control groups.

The present study did not identify the novel MERS-CoV in any subject. The reason could be that the virus was localized in some geographical areas (e.g. Middle East) and perhaps had not spread to Ghana as at the time of sampling our subjects.

In temperate countries, seasonal variations of HCoVs are discernible with most cases occurring in winter [7], [28], [29]. In China, detection of HCoV-OC43 is reported to increase in summer whereas HCoV-229E and HCoV-NL63 occurs mainly in autumn [45], [46]. Other tropical countries like Thailand have however reported peak detections of HCoV-OC43 in winter whereas HCoV-NL63 occurred frequently in autumn [29]. In sub-Saharan African countries with unique seasonal patterns such as harmattan and wet seasons, the circulation of HCoVs tends to be different. Our study recorded high detection rates of HCoVs in the harmattan and the wet seasons compared to the other seasons. Possible explanations for this include seasonal variations in host immune status to infection [47] and changes in humidity which increase viral survival in the environment [48]. In the harmattan season for instance humidity is extremely low with heavy amount of dust that could injure the respiratory system thus exposing individuals to infection [49]. Our results may be comparable to a study in Senegal that identified HCoVs in October (rainy season) [50]. Additional investigation is needed to define the seasonality of HCoVs especially in sub-Saharan African countries with tropical climates.

A comparison of the first 500 base pairs of the spike region, which is the region with most variation in the CoV genome [51], did not show differences in the strains from our study compared to reference strains from the GenBank. We also did not find variation in strains from the different communities as well as between cases and controls. This contrasts the observation of variable sequences from the spike region of HCoV-NL63 detected in three different years in the United States of America [51]. It is conceivable that connectivity on an international scale contributes to the variability of the spectrum of prevalent viruses in communities. Such connectivity may be low in the remote communities investigated here.

## Conclusion

This study has demonstrated that the same strains of HCoVs detected in the northern hemisphere circulate at high rates in a remote and rural setting in Western Africa. The general pattern of virus detection rates and virus concentrations suggest these agents to be causative for upper respiratory tract infections among older children and adults. However, in this environment with presumably low standards of personal and community hygiene, viruses are frequently detected in healthy control subjects, identifying an important caveat concerning the use of pure virological diagnostic data for clinical decision-making.
